# A practical implementation of de-Pake-ing via weighted Fourier transformation

**DOI:** 10.7717/peerj.30

**Published:** 2013-02-12

**Authors:** Marc-Antoine Sani, Daniel K. Weber, Frank Delaglio, Frances Separovic, John D. Gehman

**Affiliations:** 1 School of Chemistry, Bio21 Institute, University of Melbourne, Australia; 2 Laboratory of Chemical Physics, National Institute of Diabetes and Digestive and Kidney Diseases, National Institutes of Health, Bethesda, USA

**Keywords:** dePake, NMRPipe, Membrane perturbation, Lipid biophysics, Deuterium NMR

## Abstract

We provide an NMRPipe macro to meet an increasing need in membrane biophysics for facile de-Pake-ing of axially symmetric deuterium, and to an extent phosphorous, static lineshapes. The macro implements the development of McCabe & Wassall (1997), and is run as a simple replacement for the usual Fourier transform step in an NMRPipe processing procedure.

There has been a resurgence of interest in solid-state ^2^H and ^31^P NMR, particularly in the burgeoning area of antimicrobial peptides (Pinheiro & Watts, 1994; Tremouilhac et al., 2006; Ouellet et al., 2007; Wi & Kim, 2008; Gehman et al., 2008a; Pabst et al., 2008; Fernandez, Gehman & Separovic, 2009; Cheng et al., 2009), but also in many other research programs for which membrane-protein/peptide interactions are integral (e.g. Dufourc, Bonmatin & Dufourcq, 1989; Gehman et al., 2008b; van den Brink-van der Laan, Killian & de Kruijff, 2004; Vogel et al., 2007). Unoriented lipid vesicles are typically the most convenient sample systems used in these studies, for which broad static lineshapes are analyzed to assess perturbation by peptide. Static ^2^H NMR of enriched fluid phase acyl chain and/or head group CH_*n*_ sites produce the canonical Pake pattern (Pake, 1948), where reduction or increases in the quadrupole splitting corresponds to an increase or decrease in fluctuation in the frequency regime of 10^5^ Hz (Seelig & Seelig, 1974). Similarly, reduction or increases in the width of the ^31^P chemical shift anisotropy corresponds to an increase or decrease, respectively, in orientational order in the 10^3^ Hz regime and/or changes in the average orientation of the phospholipid headgroup (Kohler & Klein, 1977; Gehman et al., 2008b).

De-Pake-ing is one method which aids in the analysis of these static spectra, for fluid phase membranes. The procedure is a numerical transform which converts the unoriented axially symmetric static spectrum into a 0°-oriented spectrum. The de-Paked spectrum mimics that which would have been obtained if the lipids had been uniformly oriented in an aligned bilayer with the surface perpendicular to the static magnetic field (i.e. with the membrane normal oriented parallel to the static magnetic field), rather than in spherical (or spheroidal Schäfer, Mädler & Sternin, 1998) lipid vesicles. De-Pake-ing was introduced first as a computationally intensive iterative procedure (Bloom, Davis & Mackay, 1981; Sternin, Bloom & Mackay, 1983), then treated as an “ill-posed problem” using inverse theory (Whittall et al., 1989), and regularization (Schäfer, Mädler & Volke, 1995) and finally using Fourier transform (FT) of modified signal (McCabe & Wassall, 1997).

In our experience, de-Pake-ing is something of a black art within individual laboratories, including our own, owing in part to the difficulty in propagating the expertise required to control the nuanced behaviors of the procedure with freely available documentation alone. To address this problem, we offer here a simple NMRPipe (Delaglio et al., 1995) macro that can be substituted for the Fourier transform step in an otherwise identical NMRPipe script, which implements the method of de-Pake-ing by McCabe & Wassall (1997). The procedure is therefore easy to implement, and should share the same future stability as NMRPipe itself.

The theory behind “de-Paking” of axially symmetric powder patterns using weighted FT culminates in the expression (McCabe & Wassall, 1997)

(1) \begin{eqnarray*} {F}_{0}(-2 \nu )\propto \sqrt{\vert \nu \vert }(1\pm i)\text{FT}\{ g(t)\sqrt{t}\} . \end{eqnarray*}

This relates the intensity of the frequency in the oriented spectrum (*F*_0_) to the intensity of the Fourier transform of a weighted time domain signal at half the frequency on the opposite side of the spectrum. The apodization is simply multiplication by the square root of time *t*. This windowing function, unfortunately, decreases signal-to-noise in the de-Paked spectrum relative to the unoriented spectrum. The ± refers to positive and negative frequencies, i.e. the left and right halves of the spectrum, respectively. The (1 ± *i*) means the left and right halves of the spectrum come out 90° out of phase with each other. Thus the time domain can be converted to a purely absorptive de-Paked *F*_0_ spectrum by resetting spectral width and referencing parameters, reversing the spectrum, exchanging the real and inverse-imaginary channels in the right half of the spectrum, and zero-order phase correcting by −45°. The new NMRPipe macro listed and described in Fig. 1 accomplishes these steps in a straightforward fashion, and should be invoked where one would normally invoke the Fourier transform, for example:

**Table:** 

nmrPipe -in test.fid	∖
| nmrPipe -fn LS -ls 6 -sw	∖
| nmrPipe -fn GM -g2 200.0 -c 0.5	∖
| nmrPipe -fn MAC -macro $NMRTXT/dePakeFT.M -all	∖
| nmrPipe -fn PS -p0 259.0 -p1 0.0 -di	∖
| nmrPipe -ov -out depake.ft	

where LS compensates for having begun acquisition prior to the top of the solid echo, and effectively discards the early points which suffer from probe coil and (especially analog) audio filter ringing; GM is a usual apodization function; and PS applies the same frequency-independent (-p0) phase shift as required by the regular, unoriented FT spectrum. Note that the LS step requires an integral number of left-shifts, and it is too difficult to reliably adjust the frequency-dependent (-p1) phase correction in the PS step. Both of these constraints require careful optimization of the preacquisition delay in the solid echo pulse sequence (Davis, 1983).

Figure 2 shows the Pake pattern of a regular Fourier transform, and a de-Paked spectrum using the macro in Fig. 1, for data simulated using Simpson (Bak, Rasmussen & Nielsen, 2000). The NMRPipe macro works as expected: oriented spectral intensity appears in ^2^H spectra at positions corresponding to the 0° frequencies for the simulated lineshape, consistent with a splitting of 3/2 × the 10 kHz quadrupole coupling constant used for simulation, and in ^31^P spectra at the δ = 28 ppm chemical shift anisotropy value used for simulation (equivalent to the 0° edge, and corresponding to more typically quoted Δδ = −42 ppm).

**Figure 1:**
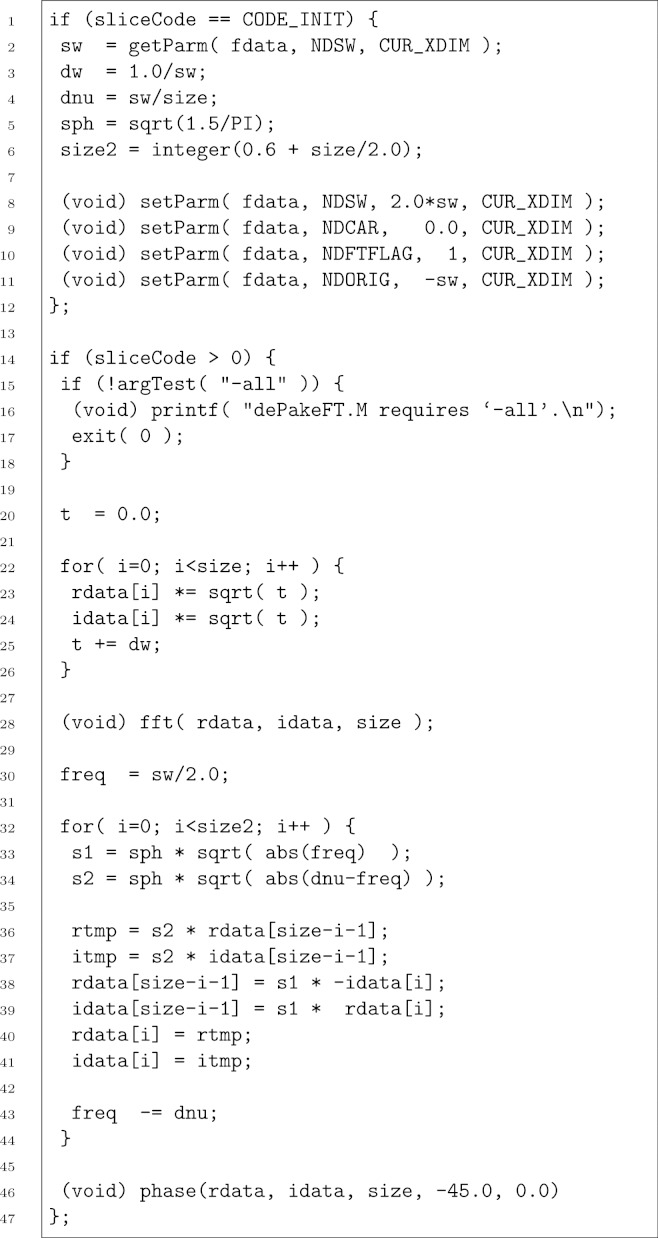
The dePakeFT macro. The first block (lines 1–12) reads the acquisition spectral width from the data header, sets a collection of constants, and resets necessary header parameters. The loop in lines 22–26 performs the $\sqrt{t}$ apodization, followed by the Fourier transformation in line 28. Lines 32–44 apply the frequency-dependent intensity scaling and differential phase corrections, followed by the uniform phase correction (line 46).

**Figure 2:**
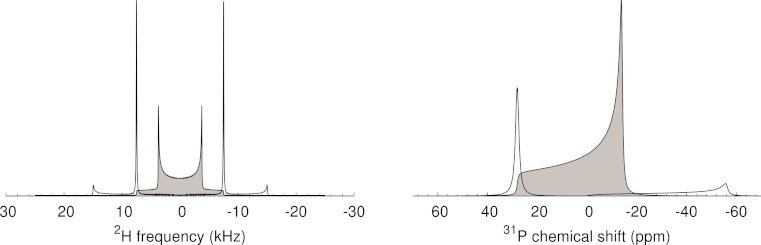
De-Pake-ing of data representative of a single CD_*n*_ site from phospholipid bilayers, and simulated with Simpson (Bak, Rasmussen & Nielsen, 2000). Regular FT static lineshapes are shown in gray, and de-Paked spectra are shown as black curves. Isotropic chemical shifts were set to zero, and the zcw4180 crystal file was used. (Left) A quadrupole splitting of 10 kHz was used in a solid echo pulse sequence simulation for ^2^H, and (Right) a chemical shift anisotropy (δ under the Haeberlen convention) of 28 ppm (Δδ = −42 ppm) was used in a simple Bloch decay simulation for ^31^P.

An artifact appears with weak intensity on the opposite side of the peak intensity in the de-Paked spectrum. This has been noted previously (McCabe & Wassall, 1997), and is a consequence of using an approximation of the asymptotic value of the underlying integral. The infinite signal-to-noise of ^31^P simulations in Figs. 2 and 3 indicate that the artifacts are attenuated approximations of the full static lineshape of each component, shifted and scaled along the frequency axis such that it spans from the center of the spectrum to ∼ 2  × δ, with opposite sign to the peak de-Paked intensity.

One of the tedious aspects of most of the de-Pake-ing methods is the need to center the first moment of the static spectrum within the spectral window, at a point with frequency of exactly zero. This requirement is less stringent for the FT method. If the carrier frequency is not centered at the isotropic chemical shift, the negation and doubling of the frequency axis involved in the de-Paking means that the oriented intensity for each side will appear at −2 × the offset compared to where it would have appeared if the carrier frequency had been centered in the Pake pattern. For example, in Fig. 2, the de-Paked (oriented) ^2^H intensity appears at ± 7.5 kHz, but the carrier offset of +3 kHz in Fig. 3 causes the de-Paked peaks to shift −6 kHz to 1.5 and −13.5 kHz. The scaling of intensities proportional to the square root of distance from the center of the spectrum (Fig. 1 lines 36–45) also causes an imbalance between the two theoretically symmetric halves of the doublet. While the frequency axis can be adjusted to center the lineshape, this is unnecessary for small shifts as the quadrupole splitting is the same with or without the offset. This is of particular benefit, as the lipid acyl CD_2_ and CD_3_ isotropic chemical shifts are slightly different. Larger offsets are, of course, a more serious concern, from an experimental set-up perspective, due to finite excitation profiles that would likely impact upon ideally uniform excitation of the very broad line.

**Figure 3:**
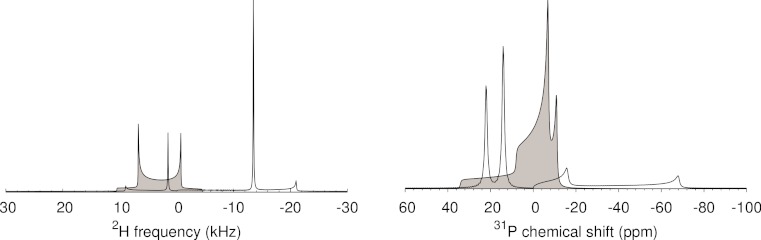
De-Pake-ing of data simulated with Simpson, using non-zero isotropic offsets. Simulations and display parameters are used as in Fig. 2, except (Left) An isotropic shift offset of 3000 Hz was used for ^2^H, and (Right) two ^31^P components are included, as may be seen under some circumstances, e.g. for a mixed phospholipid bilayer, one with a chemical shift anisotropy δ = 30 ppm (Δδ = −45 ppm) and isotropic frequency offset δ_0_ = 4 ppm, and another with δ = 10 ppm (Δδ = −15 ppm) and isotropic shift offset of δ_0_ = −2 ppm.

The same rule applies for the position of ^31^P oriented intensity when the carrier is not placed exactly on the isotropic frequency of a given phospholipid species. This is beneficial, insofar as spectra with multiple components that may differ in isotropic chemical shift can still be de-Paked. However, interpretation in this case will be more difficult, particularly for the more complicated mixtures being used to better approximate natural bilayer environments (Pinheiro & Watts, 1994; Sani, Dufourc & Gröbner, 2009). For the sake of illustration, two clearly distinct ^31^P species are shown in Fig. 3, as seen in some cases (Pukala et al., 2007). Where isotropic shift offsets are different for each species, no one frequency axis shift will satisfy all species. Consequently, some form of deconvolution is necessary to interpret the relationship between the positions of oriented intensities and the chemical shift parameters of each component line. While this may be possible, in practice, de-Paked ^31^P spectra may not always give sufficient resolution (e.g. Fig. 4). We find the maximum entropy-based analysis of slow-spinning MAS spectra (Sani, Separovic & Gehman, 2011) to be a more general solution to this problem.

**Figure 4:**
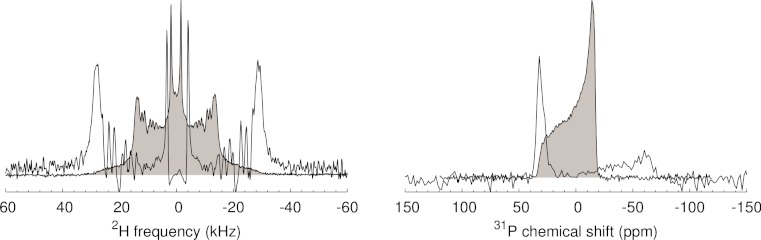
FT and de-Paked spectra of d_54_-dimyristoylphosphatidylcholine (dDMPC) multilamellar vesicles at 30 °C for: (Left) a ^2^H solid-echo pulse sequence, and (Right) Hahn-echo pulse sequence using EXORCYCLE phase cycling (Rance & Byrd, 1983).

Processing of real data (Fig. 4) indicates that the NMRPipe macro works well, and is as comparable to the Single Value Decomposition (SVD) in our experience as initially reported (McCabe & Wassall, 1997). In contrast to the SVD approach, as well as a nonlinear-least squares approach (Whittall et al., 1989), which often took an hour or two of processing and iterative optimization of parameters, processing with this macro is essentially instantaneous. For existing NMRPipe installations, the macro uses numeric parameter codes, and can be downloaded from the Gehman webpage at http://www.chemistry.unimelb.edu.au, or as a Supplementary File to this note, and placed in $NMRTXT. NMRPipe distributions of version 6.1 or greater use parameter names as in Fig. 1, and include the dePakeFT.M macro by default.

## Supplemental Information

10.7717/peerj.30/supp-1 Supplementary Information 1 Supplementary file with source code for macro This macro applies to many current versions of NMRPipe in circulation. It should be edited to output a reference to this article, if it is accepted.
